# Myasthenia Gravis: A Review

**DOI:** 10.1155/2012/874680

**Published:** 2012-10-31

**Authors:** Annapurni Jayam Trouth, Alok Dabi, Noha Solieman, Mohankumar Kurukumbi, Janaki Kalyanam

**Affiliations:** ^1^Department of Neurology, Howard University Hospital, 2041 Georgia Avenue, Washington, DC 20060, USA; ^2^Department of Physical Medicine and Rehabilitation, Howard University Hospital, 2041 Georgia Avenue, Washington, DC 20060, USA

## Abstract

Acquired myasthenia gravis is a relatively uncommon disorder, with prevalence rates that have increased to about 20 per 100,000 in the US population. This autoimmune disease is characterized by muscle weakness that fluctuates, worsening with exertion, and improving with rest. In about two-thirds of the patients, the involvement of extrinsic ocular muscle presents as the initial symptom, usually progressing to involve other bulbar muscles and limb musculature, resulting in generalized myasthenia gravis. Although the cause of the disorder is unknown, the role of circulating antibodies directed against the nicotinic acetylcholine receptor in its pathogenesis is well established. As this disorder is highly treatable, prompt recognition is crucial. During the past decade, significant progress has been made in our understanding of the disease, leading to new treatment modalities and a significant reduction in morbidity and mortality.

## 1. Epidemiology

Acquired myasthenia gravis (MG) is a relatively uncommon disorder, with prevalence rates that have increased to about 20 per 100,000 in the US population [1]. This autoimmune disease is characterized by muscle weakness that fluctuates, worsening with exertion, and improving with rest. In about two-thirds of the patients, the involvement of extrinsic ocular muscles (EOMs) presents as the initial symptom, usually progressing to involve other bulbar muscles and limb musculature, resulting in generalized myasthenia gravis (gMG). In about 10% of myasthenia gravis patients, symptoms are limited to EOMs, with the resultant condition called ocular MG (oMG) [2]. Sex and age appear to influence the occurrence of myasthenia gravis. Below 40 years of age, female : male ratio is about 3 : 1; however, between 40 and 50 years as well as during puberty, it is roughly equal. Over 50 years, it occurs more commonly in males [3]. Childhood MG is uncommon in Europe and North America, comprising 10% to 15% of MG cases. In Asian countries though, up to 50% of patients have onset below 15 years of age, mainly with purely ocular manifestations [4].

### 1.1. Historical Aspect

The first reported case of MG is likely to be that of the Native American Chief Opechancanough, who died in 1664. It was described by historical chroniclers from Virginia as *“the excessive fatigue he encountered wrecked his constitution; his flesh became macerated; the sinews lost their tone and elasticity; and his eyelids were so heavy that he could not see unless they were lifted up by his attendants… he was unable to walk; but his spirit rising above the ruins of his body directed from the litter on which he was carried by his Indians”* [2, 6]. In 1672, the English physician Willis first described a patient with “fatigable weakness” involving ocular and bulbar muscles described by his peers as “spurious palsy.” In 1877, Wilks (Guy's Hospital, London) described the case of a young girl after pathological examination as “bulbar paralysis, fatal, no disease found” [7]. In 1879, Wilhelm Erb (Heidelberg, Germany) described three cases of myasthenia gravis in the first paper dealing entirely with this disease, whilst bringing attention to features of bilateral ptosis, diplopia, dysphagia, facial paresis, and weakness of neck muscles [8]. In 1893, Samuel Goldflam (Warsaw, Poland) described three cases with complete description of myasthenia and also analyzed the varying presentations, severity, and prognosis of his cases. Due to significant contributions of Wilhelm Erb and later of Samuel Goldflam, the disease was briefly known as “Erb's disease” and later for a brief time, it was called “Erb-Goldflam syndrome” [2].

In 1895, Jolly, at the Berlin Society meeting, described two cases under the title of *“myasthenia gravis pseudo-paralytica” *[9]. The first two words of this syndrome gradually got accepted as the formal name of this disorder. He also demonstrated a phenomenon, that later came to be known as “Mary Walker effect” after she herself observed and described the same finding in 1938 [2]. This was reported as “if you stimulate one group of muscles to exhaustion, weakness is apparent in muscles that are not stimulated; an evidence of a circulating factor causing neuromuscular weakness” [10, 11].

In 1934, Mary Walker realized that MG symptoms were similar to those of curare poisoning, which was treated with physostigmine, a cholinesterase inhibitor. She demonstrated that physostigmine promptly improved myasthenic symptoms. In 1937, Blalock reported improvement in myasthenic patients after thymectomy. Following these discoveries, cholinesterase inhibitor therapy and thymectomy became standard and accepted forms of treatment for MG [12].

In 1959-1960, Nastuk et al. and Simpson independently proposed that MG has autoimmune etiology [13, 14]. In 1973, Patrick and Lindstrom were able to induce experimental autoimmune MG (EAMG) in a rabbit model using muscle-like acetylcholine receptor (AChR) immunization [15]. In the 1970s prednisone and azathioprine were introduced as treatment modalities for MG followed by plasma exchange that was introduced for acute treatment of severe MG, all supporting the autoimmune etiology [16].

### 1.2. Classification of MG

Subtypes of MG are broadly classified as follows [17]:

1. early-onset MG: age at onset <50 years. Thymic hyperplasia, usually females,
2. late-onset MG: age at onset >50 years. Thymic atrophy, mainly males,
3. thymoma-associated MG (10%–15%)
4. MG with anti-MUSK antibodies,
5. ocular MG (oMG): symptoms only affecting extraocular muscles,
6. MG with no detectable AChR and muscle-specific tyrosine kinase (MuSK) antibodies.

MG patients with Thymoma almost always have detectable AChR antibodies in serum. Thymoma-associated MG may also have additional paraneoplasia-associated antibodies (e.g., antivoltage-gated K^+^ and Ca^++^ channels, anti-Hu, antidihydropyrimidinase-related protein 5, and antiglutamic acid decarboxylase antibodies [18, 19]).

About 15% of generalized MG patients do not have anti-AChR antibodies in current lab assays. In 40% of this subgroup, antibodies to MuSK and another postsynaptic neuromuscular junction (NMJ) protein, are found. They have atypical clinical features like selective facial, bulbar, neck, or respiratory muscle weakness with occasional marked muscle atrophy and with relative sparing of the ocular muscles. Respiratory crises are more common with involvement of muscle groups like paraspinal and upper esophageal muscles. Enhanced sensitivity, nonresponsiveness, or even clinical worsening to anticholinesterase medications has also been reported. Disease onset is earlier with female predominance and thymus histology is usually normal [20]. Seronegative MG lacks both anti-AChR and anti-MuSK antibodies and forms a clinically heterogenous group with purely ocular, mild generalized, or severe generalized disease. Some patients may have low-affinity anti-AChR antibodies, nondetectable by current assays. They are essentially indistinguishable from patients with anti-AChR antibodies in terms of clinical features, pharmacological treatment response, and possibly even thymic abnormalities [21, 22].

Thymomas are frequently associated with autoimmunity. Neoplastic epithelial cells in thymomas express numerous self-like antigens including AChR-like, titin-like, and ryanodine-receptor-like epitopes [19, 23]. These antibodies react with epitopes on the muscle proteins titin and ryanodine receptor, are found mainly in association with thymoma and late-onset myasthenia gravis, and may correlate with myasthenia gravis severity. These striational antibodies are principally detected only in the sera of patients with MG and rarely found in AChR antibody-negative MG. The frequencies of striational antibodies in thymoma-associated MG patients are high. Antititin antibodies are detected in 49%–95% of thymic-associated MG, antiryanodine receptor antibodies in 70%–80%, and anti-KV1.4 (VGKC) in 40–70% of the cases [24]. Since the presence of striational autoantibodies is associated with a more severe disease in all MG subgroups, these antibodies can therefore be used as prognostic determinants in MG patients [25].

To establish the diagnosis of MG, necessary investigations include—AChR antibodies, MuSK antibodies, and CT/MR of anterior mediastinum for thymoma or thymic hyperplasia. Neurophysiological examination with repetitive nerve stimulation and jitter measurements are important in establishing the initial diagnosis, especially in patients without detectable antibodies [16].

### 1.3. Clinical Classification

The Myasthenia Gravis Foundation of America (MGFA) clinical classification divides MG into 5 main classes and several subclasses [26]. It is designed to identify subgroups of patients with MG who share distinct clinical features or severity of disease that may indicate different prognoses or responses to therapy. It should not be used to measure outcome and is as follows.

Class I MG is characterized by the following:

- any ocular muscle weakness.
- may have weakness of eye closure.
- all other muscle strengths are normal.

Class II MG is characterized by the following:

- mild weakness affecting muscles other than ocular muscles,
- may also have ocular muscle weakness of any severity.

Class IIa MG is characterized by the following:

- predominantly affecting limb, axial muscles, or both
- may also have lesser involvement of oropharyngeal muscles.

Class IIb MG is characterized by the following:

- predominantly affecting oropharyngeal, respiratory muscles, or both,
- may also have lesser or equal involvement of limb, axial muscles, or both.

Class III MG is characterized by the following:

- moderate weakness affecting muscles other than ocular muscles,
- may also have ocular muscle weakness of any severity.

Class IIIa MG is characterized by the following:

- predominantly affecting limb, axial muscles, or both,
- may also have lesser involvement of oropharyngeal muscles.

Class IIIb MG is characterized by the following:

- predominantly affecting oropharyngeal, respiratory muscles, or both,
- may also have lesser or equal involvement of limb, axial muscles, or both.

Class IV MG is characterized by the following:

- severe weakness affecting muscles other than ocular muscles,
- may also have ocular muscle weakness of any severity.

Class IVa MG is characterized by the following:

- predominantly affecting limb, axial muscles, or both,
- may also have lesser involvement of oropharyngeal muscles.

Class IVb MG is characterized by the following:

- predominantly affecting oropharyngeal, respiratory muscles or both,
- may also have lesser or equal involvement of limb, axial muscles, or both.

Class V MG is characterized by the following:

- intubation with or without mechanical ventilation, except when employed during routine postoperative management,
- the use of feeding tube without intubation places the patient in class IVb.

## 2. Pathogenesis of MG

The nerve terminals innervating the neuromuscular junctions (NMJ) of skeletal muscles arise from the terminal arborization of *α*-motor neurons of the ventral horns of the spinal cord and brain stem. The NMJ itself consists of a synaptic cleft and a 20 nm thick space that contains acetylcholinesterase (AChE) along with other supporting proteins/proteoglycans. The NMJ postsynaptic membrane has deep folds with acetylcholine receptors (AChR) tightly packed on the top of these folds.

When the nerve action potential reaches the synaptic bouton, depolarization opens voltage gated Calcium channels on the presynaptic membrane, triggering release of ACh into the synaptic cleft. The ACh diffuses into the synaptic cleft to reach postsynaptic membrane receptors where it triggers off the end-plate potential (EPP) and gets hydrolyzed by AChE within the synaptic cleft.

MuSK (muscle specific tyrosine kinase), a postsynaptic transmembrane protein, forms part of the receptor for agrin, a protein present on synaptic basal lamina. Agrin/MuSK interaction triggers and maintains rapsyn-dependent clustering of AChR and other postsynaptic proteins [27]. Rapsyn, a peripheral membrane protein on the postsynaptic membrane, is necessary for the clustering of AChR. Mice lacking agrin or MuSK fail to form NMJs and die at birth due to profound muscle weakness [2, 28].

NMJ findings that influence susceptibility to muscle weakness and MG: EPP generated in normal NMJ is larger than the threshold needed to generate the postsynaptic action potential by a measure of multiple folds. This neuromuscular transmission “safety factor” is reduced in MG patients. Reduction in number or activity of the AChR molecules at the NMJ decreases the EPP, which may be adequate at rest; but when the quantal release of ACh is reduced after repetitive activity, the EPP may fall below the threshold needed to trigger the action potential [29]. This translates as clinical muscle weakness, and when EPP, at rest is consistently below the action potential threshold, it leads to persistent weakness.

### 2.1. Effector Mechanisms of Anti-AChR Antibodies (Anti-AChR Abs)

Anti-AChR Abs affect neuromuscular transmission by at least 3 mechanisms [2]:

- complement binding and activation at the NMJ,
- antigenic modulation (accelerated AChR endocytosis of molecules cross-linked by antibodies),
- functional AChR block—preventing normal ACh to attach and act on AChR.

### 2.2. Role of CD4+ T Cells in MG

Pathogenic anti-AChR Abs are high-affinity IgGs-and their synthesis requires activated CD4+ T cells to interact with and stimulate B cells. Therefore, thymectomy, with resultant removal of AChR-specific CD4+ T cells, helps alleviate symptoms in MG patients [30]. Similarly, treatment with anti-CD4+ antibodies has also been shown to have a therapeutic impact. AIDS patients with reduction in CD4+ T cells notice myasthenic symptom improvement.

### 2.3. Role of CD4+ T-Cell Subtypes and Cytokines in MG and EAMG (Experimental Autoimmune MG)

CD4+ T cells are classified into two main subtypes: Th1 and Th2 cells. Th1 cells secrete proinflammatory cytokines, such as IL-2, IFN-*γ*, and TNF-*α*, which are important in cell-mediated immune responses. Th2 cells secrete anti-inflammatory cytokines, like IL-4, IL-6, and IL-10, which are important inducers of humoral immune responses. IL-4 further stimulates differentiation of Th3 cells that secrete TGF-*β*, which is involved in immunosuppressive mechanisms [31].

MG patients have abundant anti-AChR Th1 cells in the blood that recognize many AChR epitopes and are capable of inducing B cells to produce high-affinity anti-AChR antibodies. Th1 cells are indispensible in the development of EAMG as proven in animal models. Therapies against Th1 cytokines (TNF-*α* and IFN-*γ*) have been proven in animal models to improve EAMG symptoms [32, 33].

Anti-AChR Th2 cells have a complex role in EAMG pathogenesis. They can be protective, but their cytokines IL-5, IL-6, and IL-10 may also facilitate EAMG development [2]. CD4+ T cells that express CD25 marker and transcription factor Foxp3 are called “Tregs” and are important in maintaining self-tolerance. Tregs in MG patients may be functionally impaired and are shown to increase after thymectomy with correlated symptom improvement. Role of natural killer (NK) and natural killer T (NKT) cells in MG and EAMG: Natural killer T (NKT) cells with Tregs help in regulating anti-AChR response. Mouse models have shown inhibition of EAMG development after stimulation of NKT cells [34]. IL-18-secreted by antigen-presenting cells (APCs), stimulates NK cells to produce IFN-*γ*, which permits and enhances Th1 cells to induce EAMG. IL-18-deficient mice are resistant to EAMG, and pharmacologic block of IL-18 suppresses EAMG. MG patients have been shown to have increased serum level of IL-18, which tends to decrease with clinical improvement [35].

### 2.4. Other Autoantigens in MG

Seronegative MG patients (who lack Anti-AChR antibodies) may have anti-MuSK antibodies (up to 40% of this subgroup). Other ethnic groups or locations (e.g., Chinese and Norwegians) have lower frequencies of anti-MuSK antibodies G in seronegative MG patients. MG patients with anti-MuSK antibodies do not have anti-AChR Abs, except as reported in a group of Japanese patients [36].

Agrin/MuSK signaling pathway maintains the structural and functional integrity of the postsynaptic NMJ apparatus in the adult muscle cell. Anti-MuSK antibodies affect the agrin-dependent AChR cluster maintenance at the NMJ, leading to reduced AChR numbers. Complement-mediated damage may also be responsible for decreasing the AChR numbers at the NMJ when targeted by anti-MuSK Abs. Some human muscle cell culture studies have shown cell cycle arrest, downregulation of AChR subunit with rapsyn, and other muscle protein expression, on exposure to sera from anti-MuSK-positive MG patients [2]. Other antimuscle cell protein antibodies (e.g., antititin and antiryanodine receptor antibodies) are also postulated to have pathogenic roles in MG as discussed earlier.

## 3. Clinical Features

The cardinal feature of MG is fluctuating weakness that is fatigable, worsening with repetitive activities and improving with rest. Weakness is worsened by exposure to heat, infection, and stress [3]. The fluctuating feature distinguishes MG from other disorders that present with a similar weakness. Typically the weakness involves specific skeletal muscle groups. The distribution of the weakness is generally ocular, bulbar, proximal extremities and neck, and in a few patients, it involves the respiratory muscles. In patients with MG, the weakness is mild in 26%, moderate in 36%, and severe in 39%, associated with dysphagia, depressed cough, and reduced vital capacity [37].

Ocular muscle weakness is by far the most common initial symptom of MG, occurring in approximately 85% of patients. Generalized progression will develop in 50% of these patients in two years [37]. It presents with fluctuating ptosis and diplopia or sometimes blurry vision. Diplopia can be elicited by having the patient look laterally for 20–30 seconds resulting in eye muscle fatigue uncovering myasthenic weakness.

The ptosis can be unilateral or bilateral, fatigues with upgaze, and sustained upgaze for 30 or more seconds will usually induce it. The ptosis can be severe enough to totally occlude vision if it is bilateral. The most commonly involved extraocular muscle is the medial rectus. On clinical examination, usually more than one extraocular muscle is weak with pupillary sparing. The weakness does not follow any pattern of specific nerve or muscle involvement, distinguishing it from other disorders such as vertical gaze paresis, oculomotor palsy, or internuclear ophthalmoplegia (INO).

Bulbar muscle involvement during the course of the disorder can be seen in 60% of the patients, presenting as fatigable chewing, particularly on chewing solid food with jaw closure more involved than jaw opening [38, 39]. Bulbar symptoms with painless dysphagia and dysarthria may be the initial presentation in 15% of patients [39]. The lack of ocular involvement in these patients may be misdiagnosed as motor neuron disease. Weakness involving respiratory muscles is rarely the presenting feature in the first 2 years of onset [35]. Respiratory muscle weakness can lead to myasthenic crisis which can be life threatening, requiring mechanical ventilation and naso-gastric (NG) tube feeding. It can be precipitated by infections and certain medications such as aminoglycosides, telithromycin, neuromuscular blocking agents, magnesium sulfate, beta blockers, and fluoroquinolone antibiotics.

Involvement of the limbs in MG produces predominantly proximal muscle weakness similar to other myopathic disorders. However, the arms tend to be more often affected than the legs. Occasionally distal muscle weakness can occur in MG [40]. Facial muscles are frequently involved and make the patient appear expressionless. Neck extensor and flexor muscles are commonly affected. The weight of the head may overcome the extensors, producing a “dropped head syndrome.” Although it has become evident that the natural course of MG is general improvement in 57% and remission in 13% after the first 2 years, severe weakness can be accompanied by high mortality. Only 20% of patients remain unchanged, and mortality from the disease is 5%–9%. Only 4% of the patients who survive the first 2 years become worse. Of those who will develop generalized myasthenia, virtually, all do so by two to three years [3].

### 3.1. Diagnosis

#### 3.1.1. Tensilon (Edrophonium Chloride) Test

**Figure 1:**
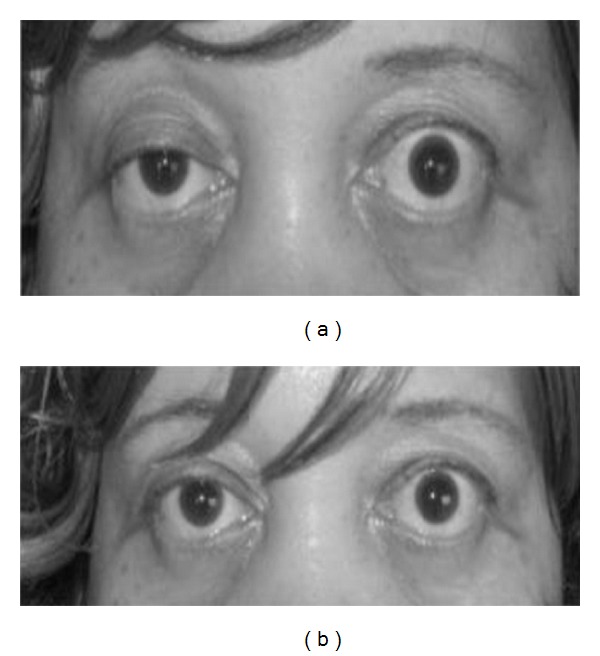
(a) Photograph of a patient with MG showing partial right ptosis. The left lid shows compensatory pseudolid retraction because of equal innervation of the levator palpabrae superioris (Herring's law). (b) Post-Tensilon test: note the improvement in ptosis (with permission from Kurukumbi et al. [5]).

Edrophonium chloride is a short-acting acetylcholinesterase inhibitor that prolongs the duration of action of acetylcholine at the NMJ. Edrophonium is administered intravenously and the patient is observed for objective improvement in muscle strength particularly the eyelid ptosis and/or extraocular muscle movement (Figure 1). Only unequivocal improvement in strength of a sentinel muscle should be accepted as a positive result. Patients must be connected to cardiac and blood pressure monitors prior to injection because of possible risk of arrhythmia and hypotension. Atropine should be available at bed side for use if an adverse event like severe bradycardia (heart rate below 37) develops. Side effects from Edrophonium include increased salivation and sweating, nausea, stomach cramping, and muscle fasciculation. Hypotension and bradycardia are infrequent and generally resolve with rest in the supine position. Tensilon test has a sensitivity of 71.5%–95% for the diagnosis of MG [41, 42].

#### 3.1.2. Ice Pack Test

The ice pack test is a non pharmacological test which could be considered in patients with ptosis when the Edrophonium test is contraindicated. It is performed by placing an ice pack over the eye for 2–5 minutes and assessing for improvement in ptosis [43].

#### 3.1.3. Electrophysiological Tests

The two principal electrophysiologic tests for the diagnosis of MG are repetitive nerve stimulation study and single fiber electromyography. Repetitive nerve stimulation tests neuromuscular transmission. It is performed by stimulating the nerve supramaximally at 2-3 Hz. A 10% decrement between the first and the fifth evoked muscle action potential is diagnostic for MG. In the absence of the decrement, exercise can be used to induce exhaustion of muscles and document decrement. The test is abnormal in approximately 75% of patients with gMG and 50% of patients with oMG [44, 45].

Single-fiber electromyography (SFEMG) is the most sensitive diagnostic test for MG. It is done by using a special needle electrode that allows identification of action potentials from individual muscle fibers. It allows simultaneous recording of the action potentials of two muscle fibers innervated by the same motor axon. The variability in time of the second action potential relative to the first is called “jitter.” In MG, the jitter will increase because the safety factor of transmission at the neuromuscular junction is reduced. SFEMG reveals abnormal jitter in 95%–99% of patients with MG if appropriate muscles are examined [44, 45]. Although highly sensitive, increased jitter is not specific for primary NMJ disease. It may be abnormal in motor neuron disease, polymyositis, peripheral neuropathy, Lambert-Eaton myasthenic syndrome (LEMS), and other neuromuscular disorders. However, it is specific for a disorder of neuromuscular transmission when no other abnormalities are seen on standard needle EMG examination [42]. The most commonly used immunological test for the diagnosis of MG measures the serum concentrations of Anti-AChR antibodies and is highly specific for myasthenia gravis [46]. False positives are rare and may occur with low titers in LEMS (5%), motor neuron disease (3% to 5%), and polymyositis (<1%).

**Figure 2:**
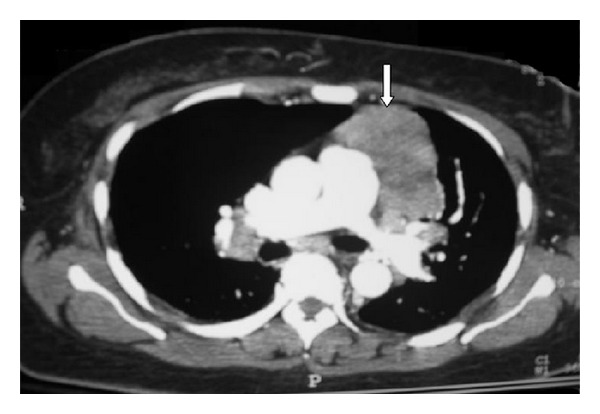
CT chest image in a patient with MG revealing large necrotic mass in the left anterior mediastinum (white arrows) and bilateral hilar lymphadenopathy (with permission from Kurukumbi et al. [5]).

 The sensitivity of this test is approximately 85% for gMG and 50% for oMG [47, 48]. Anti-AChR antibody concentrations cannot be used to predict the severity of disease in individual patients since the concentration of the antibodies does not correlate with the clinical picture. Seronegativity may occur with immunosuppression or if the test is done too early in the disease [49, 50]. As indicated above, striated muscle antibodies against muscle cytoplasmic proteins (titin, myosin, actin, and ryanodine receptors) are detected mainly in patients with thymomatous MG and also in some thymoma patients without MG [24, 51]. The presence of these antibodies in early-onset MG raises the suspicion of a thymoma. Titin antibodies and other striated muscle antibodies are also found in up to 50% of patients with late-onset and nonthymomatous MG and are less helpful as predictors of thymoma in patients over 50 years [51]. Anti-KCNA4 antibodies might be a useful marker to identify patients with thymoma but can be also seen in myocarditis/myositis [52]. Patients with gMG who are anti-AChR antibody negative should be tested for anti-MuSK antibodies which are found in approximately 40% of patients in this group. As noted, low-affinity anti-AChR antibodies binding to clustered AChRs have been found in 66% of sera from patients with seronegative gMG [53]. Whether low-affinity antibodies are present in oMG remains to be determined, but this cell-based assay might eventually provide a more sensitive diagnostic test in this subgroup. Chest CT or MRI is done in all patients with confirmed MG to exclude the presence of a thymoma (Figure 2). Iodinated contrast agents should be used with caution because they might exacerbate myasthenic weakness [54, 55]. MG often coexists with thyroid disease, so baseline testing of thyroid function should be obtained at the time of diagnosis.

**Management of Myasthenia Gravis**

Management of MG should be individualized according to patient characteristics and the severity of the disease. There are two approaches for management of MG based on the pathophysiology of the disease. The first is by increasing the amount of Acetylcholine that is available to bind with the postsynaptic receptor using an acetylcholinesterase inhibitor agent, and the second is by using immunosuppressive medications that decrease the binding of acetylcholine receptors by antibodies.

There are four basic therapies used to treat MG:

- symptomatic treatment with acetylcholinesterase inhibitors,
- rapid short-term immunomodulating treatment with plasmapheresis and intravenous immunoglobulin,
- chronic long-term immunomodulating treatment with glucocorticoids and other immunosuppressive drugs,
- surgical treatment.

### 3.2. Acetylcholinesterase Inhibitors

Acetylcholinesterase inhibitors are the first-line treatment in patients with MG. Response to treatment varies from marked improvement in some patients to little or no improvement in others. Acetylcholinesterase inhibitors are used as a symptomatic therapy and act by increasing the amount of available acetylcholine at the NMJ [56]. They do not alter disease progression or outcome. Pyridostigmine is the most commonly used drug. It has a rapid onset of action within 15 to 30 minutes reaching peak activity in about two hours. The effect lasts for about three to four hours. The initial oral dose is 15–30 mg every 4–6 hours and is titrated upwards depending on the patient's response. Adverse side effects of Pyridostigmine are mostly due to the cholinergic properties of the drug such as abdominal cramping, diarrhea, increased salivation and bronchial secretions, nausea, sweating, and bradycardia. Nicotinic side effects are also frequent and include muscle fasciculation and cramping. High doses of pyridostigmine exceeding 450 mg daily, administered to patients with renal failure, have been reported to cause worsening of muscle weakness [57].

### 3.3. Short-Term Immunomodulating Therapies

Plasma exchange and intravenous immunoglobulin have rapid onset of action with improvement within days, but this is a transient effect. They are used in certain situations such as myasthenic crisis and preoperatively before thymectomy or other surgical procedures. They can be used intermittently to maintain remission in patients with MG who are not well controlled despite the use of chronic immunomodulating drugs.

### 3.4. Plasmapheresis

It improves strength in most patients with MG by directly removing AChR from the circulation [58]. Typically one exchange is done every other day for a total of four to six times. Adverse effects of plasmapheresis include hypotension, paresthesias, infections, thrombotic complications related to venous access, and bleeding tendencies due to decreased coagulation factors [59].

### 3.5. Intravenous Immunoglobulin Therapy (IVIg)

It involves isolating immunoglobulins isolated from pooled human plasma by ethanol cryoprecipitation and is administered for 5 days at a dose of 0.4 g/kg/day, fewer infusions at higher doses are also used. The mechanism of action of IVIg is complex. Factors include inhibition of cytokines competition with autoantibodies, and inhibition of complement deposition. Interference with the binding of Fc receptor on macrophages, Ig receptor on B cells, and interference with antigen recognition by sensitized T cells are other mechanisms [60]. More specific techniques to remove pathogenic anti-AChR antibodies utilizing immunoadsorption have been developed recently, which offer a more targeted approach to MG treatment. Clinical trials showed significant reduction of blocking antibodies with concomitant clinical improvement in patients treated with immunoadsorption techniques [61].

IVIg is considered to be safe but rare cases of complications do occur such as thrombosis due to increased blood viscosity and other complications related to large volumes of the infused preparation [62].

Compared to plasma exchange, IVIg is similar in terms of efficacy, mortality, and complications [63]. However, plasma exchange (PLEX) has considerable cost advantages over IVIg with a cost benefit ratio of 2 : 1 for treatment of myasthenia gravis [64].

### 3.6. Long-Term Immune Therapies

The goal of immune-directed therapy of MG is to induce a remission or near remission of symptoms and maintain it.

### 3.7. Corticosteroids

Corticosteroids were the first and most commonly used immunosuppressant medications in MG. Prednisone is generally used when symptoms of MG are not adequately controlled by cholinesterase inhibitors alone. Good response can be achieved with initial high doses and then tapering it to the lowest dose to maintain the response. Temporary exacerbation can occur after starting high doses of prednisone within the first 7–10 days which can last for several days [65, 66]. In mild cases, cholinesterase inhibitors are usually used to manage this worsening. In cases known to have severe exacerbations, plasma exchange or IVIg can be given before prednisone therapy to prevent or reduce the severity of corticosteroid-induced weakness and to induce a more rapid response. Oral prednisone might be more effective than anticholinesterase drugs in oMG and should therefore be considered in all patients with oMG [67, 68].

### 3.8. Nonsteroidal Immunosuppressive Agents

Azathioprine, a purine analog, reduces nucleic acid synthesis, thereby interfering with T-and B-cell proliferation. It has been utilized as an immunosuppressant agent in MG since the 1970s and is effective in 70%–90% of patients with MG [65]. It usually takes up to 15 months to detect clinical response. When used in combination with prednisone, it might be more effective and better tolerated than prednisone alone [69]. Adverse side effects include hepatotoxicity and leukopenia [70].

Mycophenolate mofetil selectively blocks purine synthesis, thereby suppressing both T-cell and B-cell proliferation. Widely used in the treatment of MG, its efficacy in MG was actually suggested by a few non-randomized clinical trials [71, 72]. 

The standard dose used in MG is 1000 mg twice daily, but doses up to 3000 mg daily can be used. Higher doses are associated with myelosuppression, and complete blood counts should be monitored at least once monthly. The drug is contraindicated in pregnancy and should be used with caution in renal diseases, GI diseases, bone marrow suppression, and elderly patients [73].

Cyclophosphamide administered intravenously and orally is an effective treatment for MG [74]. More than half of the patients become asymptomatic within 1 year of treatment. Undesirable side effects include hair loss, nausea, vomiting, anorexia, and skin discoloration, which limit its use to the management of patients who do not respond to other immunosuppressive treatments [2].

Cyclosporine blocks the synthesis of IL-2 cytokine receptors and other proteins critical to the function of CD4+ T cells. Cyclosporin is used mainly in patients who do not tolerate or respond to azathioprine. Large retrospective studies have supported its use as a steroid-sparing agent [75].

Tacrolimus has been used successfully to treat MG at low doses. It has the theoretical advantage of less nephrotoxicity than cyclosporine. However, there are more controlled trial data supporting the use of cyclosporine. Like other immunosuppressive agents, Tacrolimus also has the potential for severe side effects [2].

MG patients resistant to therapy have been successfully treated with cyclophosphamide in combination with bone marrow transplant or with rituximab, a monoclonal antibody against the B cell surface marker CD20 [76].

Etanercept, a soluble and a recombinant tumor necrosis factor (TNF) receptor blocker, has also been shown to have steroid-sparing effects in studies on small groups of patients [2, 77].

### 3.9. Surgical Management

**Thymectomy**

Surgical treatment is strongly recommended for patients with thymoma. The clinical efficacy of thymectomy in other situations has been questioned because the evidence supporting its use is not solid. Surgical treatment is strongly recommended for patients with thymoma. The benefit of thymectomy evolves over several years. Thymectomy is advised as soon as the patient's degree of weakness is sufficiently controlled to permit surgery. Patients undergoing surgery are usually pretreated with low-dose glucocorticoids and IVIg. Thymectomy may not be a viable therapeutic approach for anti-MuSK antibody-positive patients because their thymi lack the germinal centers and infiltrates of lymphocytes that characterize thymi in patients who have anti-AChR antibodies. This supports a different pathologic mechanism in anti-MuSK Ab-positive and anti-AChR Ab-positive MG [78, 79]. Most experts consider thymectomy to be a therapeutic option in anti-AChR Ab-positive gMG with disease onset before the age of 50 years [2].

### 3.10. Rehabilitation

A rehabilitation program in combination with other forms of medical treatment can help relieve symptoms and improve function in MG. The primary goal is to build the individual's strength to facilitate return to work and activities of daily living. The intensity and progression of the exercise depend on the stage of the disease and overall health. An interdisciplinary approach including neuromuscular medicine, physical medicine and rehabilitation, and respiratory therapy is recommended. Physical therapy is beneficial for long-term restoration of muscle strength. Graded strengthening exercises help the individual remain as functional as possible. Occupational therapy helps the individual adapt to new ways of performing daily living tasks using energy conservation and compensatory techniques. There is speech therapy for training of esophageal speech following a tracheostomy. Vocational counseling may be needed if the current job requirements cannot be met. Psychological interventions to cope with the illness may be necessary.

## Abbreviations

AChE: Acetylcholine esterase enzyme
AChR: Acetylcholine receptor
Anti-AChR abs: Antiacetylcholine receptor antibodies
APCs: Antigen-presenting cells
EAMG: Experimental autoimmune myasthenia gravis
EOMs: Extraocular muscles
gMG: Generalized myasthenia gravis
Hz: Hertz
Interferon: Interferon
IL: Interleukin
IVIg: Intravenous immunoglobin
LEMS: Lambert Eaton myasthenic syndrome
MuSK: Muscle-specific tyrosine kinase
MG: Myasthenia gravis
MGFA: Myasthenia gravis foundation of America
NK cells: Natural killer cells
NKT cells: Natural killer T cells
NMJ: Neuromuscular junction
oMG: Ocular myasthenia gravis
SFEMG: Single-fiber electromyography
Th cell: T helper cell
Tregs: T regulator cells
TGF: Tissue growth factor
TNF: Tissue necrosis factor.
